# The Replacement of five Consecutive Amino Acids in the Cyt1A Protein of *Bacillus thuringiensis* Enhances its Cytotoxic Activity against Lung Epithelial Cancer Cells

**DOI:** 10.3390/toxins10030125

**Published:** 2018-03-16

**Authors:** Kavita Nair, Ahmad Iskandarani, Roda Al-Thani, Ramzi Mohammad, Samir Jaoua

**Affiliations:** 1 Biological & Environmental Sciences Department, College of Arts and Sciences, Qatar University, P.O. Box 2713 Doha, Qatar; kavita.nair@qu.edu.qa (K.N.); ralthani@qu.edu.qa (R.A.-T.); 2 Interim Translational Research Institute, Hamad Medical Corporation, P.O. Box 3050 Doha, Qatar; aiskandarani@hamad.qa; 3 Karmanos Cancer Institute, Wayne State University, 732 HWCRC, 4100 John R., Detroit, MI 48201, USA; mohammar@karmanos.org

**Keywords:** *Bacillus thuringiensis* subsp. *israelensis*, Novel Cyt1A, cytotoxicity, lung epithelial cancer cell line, protein modelling

## Abstract

Cyt1A protein is a cytolytic protein encoded by the *cyt* gene of *Bacillus thuringiensis* subsp. *israelensis* (Bti) as part of the parasporal crystal proteins produced during the sporulation. Cyt1A protein is unique compared to the other endotoxins present in these parasporal crystals. Unlike δ-endotoxins, Cyt1A protein does not require receptors to bind to the target cell and activate the toxicity. It has the ability to affect a broad range of cell types and organisms, due to this characteristic. Cyt1A has been recognized to not only target the insect cells directly, but also recruit other endotoxins by acting as receptors. Due to these mode of actions, Cyt1A has been studied for its cytolytic activity against human cancer cell lines, although not extensively. In this study, we report a novel Cyt1A protein produced by a Bti strain QBT229 isolated from Qatar. When tested for its cytotoxicity against lung cancer cells, this local strain showed considerably higher activity compared to that of the reference Bti and other strains tested. The possible reasons for such enhanced activity were explored at the gene and protein levels. It was evidenced that five consecutive amino acid replacements in the β8 sheet of the Cyt1A protein enhanced the cytotoxicity against the lung epithelial cancer cells. Such novel Cyt1A protein with high cytotoxicity against lung cancer cells has been characterized and reported through this study.

## 1. Introduction

*Bacillus thuringiensis* (Bt) is a Gram-positive, spore forming, aerobic soil bacterium found in different environments [1]. It belongs to the six-member group of *Bacillus cereus*, including others like *Bacillus anthracis*, *Bacillus cereus*, *Bacillus weihenstephanensis*, *Bacillus pseudomycoides*, and *Bacillus mycoides.* The factor differentiating Bt from the others is that it forms parasporal protein crystals during the sporulation stage [2]. The parasporal crystals are made up of many insecticidal proteins called δ-endotoxins including Cry toxins and cytolytic proteins called Cyt toxins [3]. It has been demonstrated that the Cry toxins are specifically insecticidal to larvae of many insect orders including Lepidoptera, Diptera, Coleoptera, Hymenoptera, Isoptera, etc., [4,5]. The specificity of the Cry toxins towards each order of insects is based on its mode of action, where the Cry toxins require specific binding receptors in the insect midgut to bind. The Cyt proteins, on the other hand, do not require receptors and can initiate the lysis of insect gut epithelial cells without specific binding [6]. Bt Cry toxins are very specific to their host families and, hence, have been recognized as the most efficient and sustainable biological alternative to the harmful chemical insecticides [7].

Apart from the insecticidal activity, Bt is also known for its antimicrobial, antifungal, and anti-cancer properties [8,9,10,11]. The anticancer properties of Bt is being widely studied today as it has high cytotoxic activity towards cancer cell lines without affecting the normal cells at the same levels of treatment. The anticancer property of Bt is attributed to two factors: Parasporins and Cytolytic proteins (Cyt) [12]. Parasporins are Cry like proteins with less that 25% amino acid sequence homology with the known Cry toxins. Like Cry toxins, Parasporins also act specifically to their target cancer cell lines as they require specific binding receptors on the cell lines for their activity [13,14,15]. They have now been classified into six groups: Parasporin 1 to Parasporin 6 [16,17,18].

Bt has three *cyt* gene families comprising of 38 *cyt* genes (http://www.lifesci.sussex.ac.uk/home/Neil_Crickmore/Bt/). Cyt proteins affect a wider range of target cells and organisms as they do not require any specific receptors [19]. They are known to have much lower insecticidal or cytotoxic activity than Cry or Parasporins [20]. They are also very useful for their ability to synergize other proteins, like Cry4 and Cry11 [21,22]. Therefore, through their synergistic action, Cyt proteins enhance the Bt insecticidal and cytotoxic activities.

In the recent years, the lack of efficient treatment strategies and the ever-increasing rates of cancers, have pushed the researchers to study the anticancer property of Bt intensively. In this study, Bt isolates collected locally from Qatari soil were explored for their cytotoxic activity. *Bacillus thuringiensis* subsp. *israelensis* (Bti) H14 and *Bacillus thuringiensis* subsp. *kurstaki* HD1 were used as reference strains. The isolates used in this study were characterized as Bti-like, based on their proteomic and genomic studies [23]. In spite of the similarity between the Bti and Qatari Bti-like isolates, the cytotoxic activity observed among the latter were considerably higher against a lung cancer cell line. After eliminating the possible presence of *parasporin* genes, it was found that the amino acid sequence of the Cyt protein from Qatari Bti strain QBT229 differed considerably from that of the reference H14 along the β8 sheet of the Cyt protein. The β8 sheet is involved in two processes: binding to the lipid bilayer of the target cell membrane and act as a receptor for Cry11 protein. This is a first study where a novel Cyt1A protein has been reported with amino acid replacements in the β8 sheet which enhances the cytotoxicity of the Bt crystal proteins against lung cancer cells.

## 2. Results

### 2.1. Determination of Cytotoxicity of Qatari Bt Strain Proteins against Lung Cancer Cells

**Figure 1:**
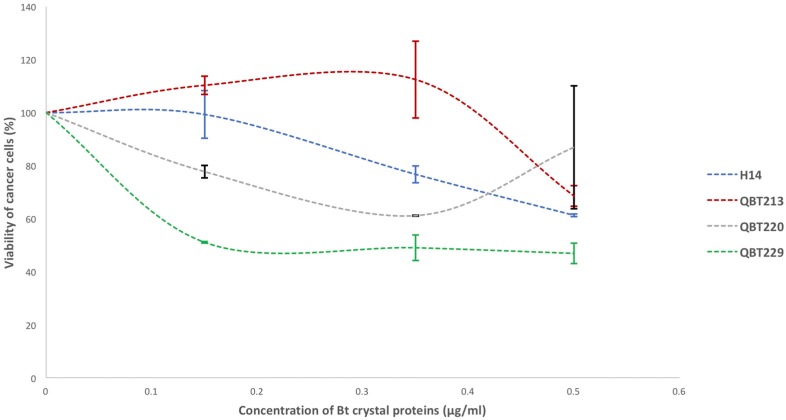
Cytotoxic effects of local Bt subsp. *israelensis* strains against lung epithelial cancer cells (NCI-H1975). Calculated viability of cells plotted against the concentration of endotoxin proteins (treatment).

The standard MTT assay for quantifying the cytotoxic activity of the Bt proteins was performed for this study with some modifications. After 72 h of incubation with solubilized and activated Bt strain proteins, the cells were treated with MTT reagent for three hours. This allowed the reagent to be converted to formazan by the live cells. The quantity of the formazan formed in each well corresponds to the number of live cells in each well. Hence, the untreated well represented 100% viability. The formazan was quantified by solubilizing in dimethyl sulfoxide (DMSO) and checking the absorbance by the purple dye formed during the process. The three Qatari Bti strains QBT213, QBT220 and QBT229 showed varying degrees of cytotoxic activity after 72 h incubation (Figure 1). However, QBT229 showed the highest cytotoxicity. In fact, while the reference strain Bt subsp. *israelensis* H14 and other local Bt subsp. *israelensis* strains QBT213 and QBT220 showed cell viability of more than 60% even at the highest concentration of solubilized Bt proteins tested (0.5 µg/mL), the Bt subsp. *israelensis* QBT229 could kill more than 50% of cancer cells even at the lowest concentration of solubilized Bt proteins tested (0.15 µg/mL).

### 2.2. Investigation of Genes Encoding Endotoxins, Parasporin, and Cyt Proteins

**Figure 2:**
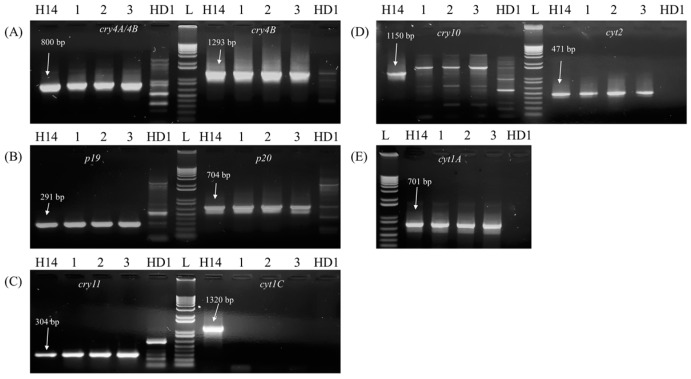
Electrophoresis of the amplified PCR products obtained for each δ-endotoxin gene being explored, Lanes L represent 1 kb plus ladder (100 bp, 200 bp, 300 bp, 400 bp, 500 bp, 650 bp, 850 bp, 1 kb, 1.65 kb, 2 kb, 5 kb, and 12 kb), Lanes 1, 2, and 3 represent QBT213, QBT220, and QBT229 respectively, Lanes H14 and HD1 represent references Bt subsp. *israelensis* and Bt subsp. *kurstaki* respectively: each panel represents PCR amplification results for (**A**) 800 bp of *cry4A*/*4B* and 1293 bp of *cry4B*; (**B**) 291 bp of *p19* & 704 bp of *p20*; (**C**) 304 bp of *cry11* & 1320 bp of *cyt1C*; (**D**) 1150 bp of *cry10* & 471 bp of *cyt2*; and (**E**) 701 bp of *cyt1A.*.

To understand the difference in cytotoxic activity between the QBT strains and the reference strain Bti H14, PCR screenings were performed to identify the types of genes they might be carrying. For these screenings, primer sets were designed or adopted from the published articles to detect the presence of the genes of interest (Table 1). Among the endotoxin coding genes carried by reference H14, the Qatari Bti-like isolates showed the presence of *cry4A/4B*, *cry11*, *p19*, and *p20*, except *cry10* (Figure 2). The PCR amplification with the cry10 primer sets produced a PCR product of about 2 kb, instead of the expected 1.1 kb given by the reference (Figure 2D). This PCR product was purified from the gel and sequenced by Sanger sequencing and the sequence had no resemblance to any known endotoxin genes. It was concluded that the three Qatari Bt subsp. *israelensis* strains lack the gene *cry10*.

Among the Cyt family of proteins, Qatari isolates showed the presence of *cyt1A* and *cyt2A* genes, but gave no PCR product with primer sets designed for *cyt1C* gene (Figure 2). Among the parasporin genes, neither the reference (as expected) nor the Qatari isolates gave any PCR products for any of the primer sets designed for parasporin genes (data not shown).

### 2.3. Investigation of the cyt1A Gene of Qatari Bt subsp. israelensis Strain QBT229

QBT229 showed the highest cytotoxic activity among the Qatari isolates (Figure 1). It showed a LC50 of about 0.15 µg/mL as opposed to the reference H14 that showed 60% viability even at highest concentration tested (0.5 µg/mL). Hence, the *cyt1A* gene sequence of QBT229 (accession number: MG708177) was compared with that of *cyt1A* gene of the reference Bt subsp. *israelensis* H14. The comparison between the sequences revealed at least six nucleotide differences among the sequences.

### 2.4. Translation and Amino Acid Sequence Alignment to Study the Cyt1A Protein

**Figure 3:**
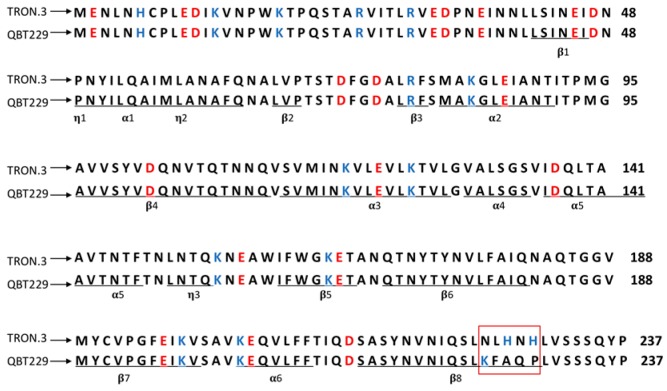
Amino acid alignment of Cyt1A protein sequence from QBT229 and the template TRON.3 representing the Cyt1A sequence and model for Cyt1A protein from *Bacillus thuringiensis* subsp. *israelensis*; red box represents the region of β8 sheet with amino acid replacements; amino acids highlighted in red have negative charges and the amino acids highlighted in blue have the positive charge; amino acids included in secondary structures like β sheets, α helix, and η are marked in the template.

The DNA sequence of *cyt1A* gene from QBT229 (accession number: MG708177) was translated into amino acid sequence and aligned with published cyt1A protein sequence using in silico tools. The alignment of Cyt1A amino acid sequences of QBT229 and that of reference H14 revealed six amino acid replacements (Figure 3). At position 225, Lysine has been replaced by Asparagine, at position 226 Phenylalanine has been replaced by Leucine, at position 227 Alanine has been replaced by Histidine, at position 228 Glutamine has been replaced by Asparagine and at position 229 Proline has been replaced by another Histidine.

### 2.5. Chemical Differences Due to Amino Acid Replacements by Protein Modelling

**Figure 4:**
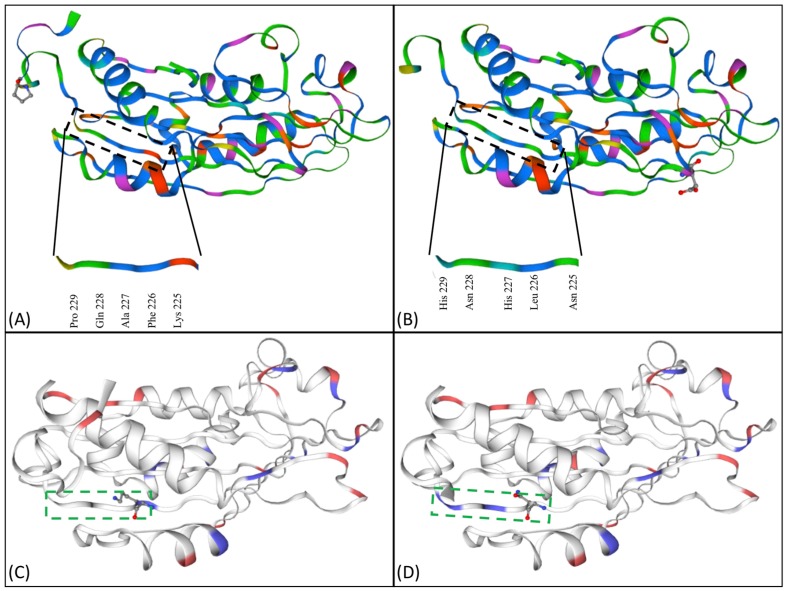
Protein modeling and structural and chemical homology comparisons between Cyt proteins. The ClustalX protein model of the Cyt protein expressed by (**A**) Bt subsp. *israelensis* H14 and (**B**) Qatari Bt subsp. *israelensis* QBT229, showing the amino acid replacements and chemical differences in the region; the positive (blue) and negative (red) charges owing to the amino acids in the Cyt protein of (**C**) Bt subsp. *israelensis* H14 and (**D**) Qatari Bt subsp. *israelensis* QBT229.

When the region of the protein structure with amino acid replacements (position 225 to 229) were checked, no structural changes were observed in the region (Figure 4A,B). The region was further studied based on possible chemical changes, in order to understand the effect of these changes on the cytotoxicity. In this region, QBT229 has four polar and positively charged amino acids (2× Asn, 2× His) and one hydrophobic amino acid (Leu) that have replaced three hydrophobic amino acids (Phe, Ala, Pro), one polar amino acid (Gln) and one positively-charged amino acid (Lys) (Table 2). Cyt1A protein consists of eight β sheets and six α helices. The region consisting of amino acids from position 225 to 229 forms part of β8 sheet (Figure 3). All these changes in QBT229 have made this region comparatively positively charged and polar (Figure 4C,D, Table 2).

## 3. Discussion

The parasporal crystal proteins of Qatari Bt subsp. *israelensis* strains were tested against lung epithelial cancer cells to quantify their cytotoxic effects by MTT assay. It was found that the cytotoxic activities exhibited by Qatari Bt subsp. *israelensis* strains were much higher than the Bti strain H14. From previous research, it is known that the cytotoxicity by Bt is attributed to either the anticancer proteins called Parasporins or the cytolytic proteins called Cyt [12]. PCR with whole DNA extract from all the Qatari Bti-like isolates and the reference failed to amplify any of the *parasporin* genes. This was expected as the insecticidal activity due to endotoxins and the anticancer property due to parasporins are known to be mutually exclusive among the Bt strains [24,25]. However, the lack of *parasporin* genes warranted further investigation of the *cyt1A* gene. It was found that Qatari Bti like isolates lacked two of the genes that the Bti carried: *cry10* and *cyt1C*. But, they all gave the expected amplification for the PCRs carried out with primers detecting other genes such as *cry4A/cry4B*, *cry11*, *cyt1A*, *cyt2B*, *p19*, and *p20*. Among these genes, *cyt1A* gene encodes Cyt1A protein that is known to specifically act on cancer cells [26,27]. As the reference and Bti like isolates showed the presence of *cyt1A* gene, the cytotoxicity could be attributed to its encoded Cyt protein.

To explore the difference in cytotoxic activity among the reference and local isolates, the Cyt protein of one local isolate QBT229 with the highest cytotoxicity was compared with that of the reference. The full *cyt1A* gene was amplified by PCR and the amplified product was gel purified for sequencing by the Sanger’s method. The *cyt1A* gene nucleotide sequence (accession number: MG708177) showed considerable differences when compared to the published sequence of *cyt1A* gene from Bti H14 strain. The sequence was translated to amino acid sequence and five consecutive amino acid replacements (position 225 to 229) were detected towards the C terminal of the protein. The sequence was fed to the SWISS-MODEL to render the 3D model of the consequent protein to see if there were any structural differences. When compared, no conformational changes were detected between the Cyt1A structure of reference and that of QBT229 (Figure 4A,B). When different filters for chemical changes in the region were applied to the software, it was found that the studied region of QBT229 gained positive charges and has become more polar than that of the reference (Table 1). Among these five replacements, four amino acids are polar with a positive charge.

It is known that these amino acids form a part of the β8 sheet of the Cyt1A protein (Figure 3). The β sheets of Cyt1A protein are involved in membrane pore formation, in order to activate the cytotoxic effect against the target cell. A change in these β sheets would in turn affect the cytotoxicity of the Cyt1A protein. Additionally, the β8 sheet is known to be involved in synergizing other endotoxins like Cry11. Cry11 is one of the insecticidal endotoxins carried by the mosquitocidal Bt strains, that require specific binding receptors on the membrane of the target cell to act as a toxin. Cry11 does not affect the human cell lines, except when Cyt1A protein is present along with it. Cyt1A binds to the lipid bilayer of the cell membrane and acts as a receptor for the Cry11 protein. Specifically, the three amino acids at the positions K198, E204, and K225 of the Cyt1A protein interact with the amino acids at the positions S259 and E266 of the Cry11 protein [28]. Thus, the interaction takes place between the charged amino acids of Cyt1A (K198, E204, and K225), and one polar (S259) and one charged amino acid (E266) of the Cry11 protein.

In case of the Cyt1A protein of QBT229, it was found that the K225 (Lysine) of β8 sheet has been replaced by N225 (asparagine). In this case, the interactions between Cyt1A and Cry11 protein of QBT229 take place between two charged (K198, E204) and one polar (N225) amino acid of Cyt1A and one charged (E266) and one polar amino acid (S259) of Cry11. Hence, it was postulated that the interactions were enhanced because of the presence of polar molecules on both sites.

Additionally, the overall β8 sheet has been changed chemically due to the five amino acid replacements (Figure 4). The amino acids K225, F226, A227, Q228, and P229 of the Cyt1A has been replaced by N225, L226, H227, N228, and H229 in the case of QBT229 Cyt1A protein. The positively-charged polar amino acids in the position 225, 227, 228, and 229 increases the ability of these amino acids to interact with the polar lipids. These interactions are enhanced because of the availability of more amino acids in this region that could be involved in hydrogen bonding, which is the characteristic of polar amino acids.

In other words, the amino acid replacements seen in Cyt1A protein of QBT229 would most probably increase the affinity between the β8 sheet and the lipid polar heads, increasing the binding affinity between the Cyt1A protein and the membrane, thereby enhancing its cytotoxicity. The specific amino acids replacement at position 225 enhances its ability to recruit the Cry11 protein to the membrane, thereby contributing to the cytotoxicity.

## 4. Conclusions

The cytotoxic activity of three Qatari Bt subsp. *israelensis* strains was quantified and compared to that of the reference Bt subsp. *israelensis* H14 against lung cancer epithelial cells NCI-H1975. It was observed that the cytotoxic activity among these Qatari Bt strains were higher compared to the reference strain H14. The presence of anticancer proteins called Parasporins was eliminated. Hence, it was postulated that the cytotoxic activity shown against the NCI-H1975 cell lines could be caused by the Cyt1A protein. The full *cyt1A* gene sequence of the Qatari Bt subsp. *israelensis* strains showing the highest cytotoxic activity (QBT229), showed several differences when compared to the *cyt1A* gene sequence of the reference H14. To understand the possible significances of the nucleotide replacements, the amino acid sequences were studied and it was found that there are five amino acid replacements. These replacements did not bring about any structural differences but, chemically, the region has changed. The Cyt1A protein of QBT229 had four positively-charged polar amino acids instead of one polar and one positively-charged amino acid in the Cyt1A protein of reference Bti. The polarity and the charge of the region with amino acid replacements were enhanced. As this particular region is involved in membrane binding via lipid polar heads, it was concluded that the polar amino acids of β8 sheet of QBT229 has a better binding ability compared to the reference Cyt1A. In addition, the amino acid replacement at position 225 enhances its ability to act as receptor to the Cry11 protein. In our study, it was shown for the first time a novel Cyt1A protein which has amino acid replacements that enhance its cytotoxic activity against lung cancer cells without losing its structural homology.

## 5. Materials and Methods

### 5.1. Bt Isolates and Culture Conditions

Four *Bacillus thuringiensis* strains were used in the present study; H14 (Bti reference strain) and three local Bt subsp. *israelensis* isolates from Qatar environment. The Bt isolates were grown on nutrient-rich, low sodium Luria Bertini (LB) media at 30 °C. The pure cultures were stored in the form of spore-crystal mixture on T3 sporulation media [29]. The T3 agar plates inoculated with the Bt isolates were incubated at 30 °C for 96 h for complete sporulation. For all the experiments conducted, the spore-crystal mixtures were inoculated and incubated on LB agar plates overnight at 30 °C to obtain fresh cultures.

### 5.2. Parasporal Crystal Protein Purification and Solubilisation

During sporulation, Bt produces spores and the associated parasporal protein crystals are released into the sporulation broth. For the cytotoxicity assays, it is necessary to separate these crystals from the spores before activation. The separation was done by solubilizing the crystals at high pH maintained by 50 mM Na_2_CO_3_ for one hour at room temperature [30]. Once the proteins are solubilized, they were separated from the spores by centrifugation at 10,000 rpm for 10 min at 40 °C. The high pH of the solution was then adjusted to around 7, as the high pH affects the activity of proteases used for the next step of activation. The supernatant containing all the crystal proteins were then activated by trypsin at a final concentration of 15 µg/mL for 1 h at 37 °C. This activation is an essential step to convert the pro-toxins from the crystals to toxins. For the quantitative assay, the proteins were quantified using the spectrophotometer and extrapolating the values on the standard curve of optical densities (O.D) of bovine serum albumin by Bradford’s method. The quantified proteins were used as treatment to be added according to the different concentrations decided for the assay: 0.15 µg/mL, 0.35 µg/mL, and 0.5 µg/mL. Each test was triplicated to obtain statistically relevant results.

### 5.3. Cancer Cell Line and Culture Conditions

The cell line chosen was the adherent lung cancer epithelial cells from ATCC called NCIH1975 [H1975, H1975] (ATCC^®^ CRL5908™). The cells were maintained in ATCC-formulated RPMI-1640 medium added with 10% fetal bovine serum (FBS), 100 µg/mL Streptomycin and 100 IU/mL penicillin. The cells were grown and maintained in humidified 5% CO_2_ incubator at 37 °C.

### 5.4. Quantitative Cytotoxic Bioassay

The cytotoxicity was determined by the cell proliferation assay called MTT assay [31]. The activity was quantified by comparing the percentage of viable lung cancer epithelial cells remaining after the treatment in the treated wells and untreated wells of cells. Each of the five concentrations were repeated thrice. Each well of the 96-well microtiter plates were seeded with 5000 cells and incubated overnight for attachment. Then the media from each well was replaced with fresh media containing fixed concentrations of proteins for treatment. The treatments were carried out for 72 h. After 72 h, 100 µL of MTT reagent was added to each well and incubated for three hours at 37 °C. 50 µL of DMSO was added to each well and absorbance was calculated using the plate reader (Tecan, Switzerland). The cytotoxicity was calculated by comparing the cell viability observed in the treated wells to that of the untreated wells based on their respective absorbance.

### 5.5. Isolation of Plasmid DNA

The total plasmid DNA was isolated from fresh bacterial cultures by alkaline lysis method and purified by alcohol precipitation [32]. The cells were lysed by high pH maintained by NaOH and lysozyme digestion at 37 °C. The cell debris and proteins were separated from the nucleic acids by phenol: chloroform: isoamyl alcohol (25:24:1) precipitation. The nucleic acids were purified by ethanol precipitation overnight at −20 °C. The RNA molecules were digested by RNase enzyme (4 µg/mL) at 37 °C for one hour to obtain plasmid DNA.

### 5.6. Exploration of Endotoxin and Parasporin Encoding Genes

The primers were designed or adopted from published articles for the δ-endotoxin genes, *parasporin* genes and cytolytic (*cyt*) genes (Table 2). Polymerase chain reactions (PCR) were conducted with these primer sets for each plasmid sample. The reactions were carried out with Qiagen Taq PCR Master Mix Kit (Germany). The cycles for the amplifications included one denaturation step for 5 min at 95 °C followed by 35 cycles consisting of 1 min denaturation at 95 °C, 1 min annealing at the appropriate temperature and 1 min 30 s polymerization at 72 °C. The final extension was set at 72 °C for 7 min. A gel of 1.2% agarose stained with ethidium bromide was used for the visualization of the PCR products by gel electrophoresis.

### 5.7. Gel Purification and DNA Sequencing of PCR Products

Forty microliters of the PCR product to be sequenced was loaded on a 1% agarose gel and the gel electrophoresis was carried out for one hour at 50 V. The DNA band to be sequenced was cut from the gel by visualizing the same on a gel dock with UV light. The DNA was purified from the gel using QIAquick gel extraction kit from Qiagen (Hilden, Germany), as per the manual instructions. The purified product was then sequenced by Sanger sequencing.

### 5.8. Translation, Alignment and Comparison of Amino Acid Sequences

The DNA sequence obtained from sequencer was translated into amino acid sequence in silico using the translate tool of ExPasy available on Swiss Institute of Bioinformatics research portal. The sequence was aligned to the published sequence of the *cyt* gene using the pBLAST tool on the National Centre for Biotechnology Information (NCBI) website.

### 5.9. In Silico Structural Homology Comparison of Cyt Proteins

To understand the structural and/or chemical differences among the functional Cyt protein structures of QBT229 and the reference Bt subsp. *israelensis* H14, their respective amino acid sequences were used to find the in silico structure of the proteins by SWISS-MODEL software [38]. As a reference for the protein modelling, the 3D structure of the Cyt1A1 protein (TRON.3) from *Bacillus thuringiensis* subsp. *israelensis* was used. Cohen et al. [39] had modelled the structure of Cyt1A1 protein by X-ray crystallography with a resolution of 2.19 Å. The structures of the two Cyt proteins were compared for any conformational changes due to amino acid replacements. The structures were also compared chemically by applying the different filters of the software including charged regions, hydrophobic regions, polar regions, etc.

## Figures and Tables

**Table 1:** The list of amino acid replacements seen in Qatari Bt subsp. *israelensis* QBT229 Cyt1A protein compared to the reference Bti H14.

Amino Acid Positions	Bt subsp. *israelensis* H14	Qatari Bt subsp. *israelensis* QBT229
225	Lysine (+) (Charged)	Asparagine (+) (Polar)
226	Phenylalanine (Hydrophobic)	Leucine (Hydrophobic)
227	Alanine (Hydrophobic)	Histidine (+) (Polar)
228	Glutamine (Polar)	Asparagine (+) (Polar)
229	Proline (Hydrophobic)	Histidine (+) (Polar)

**Table 2:** List of primers used in this study to explore the Bt subsp. *israelensis* genes.

Sr. No	Genes	Primer Pairs	Sequences	Amplicon Size	References
1	*cry4A*, *cry4B*	Dip1A	5′ CAAGCCGCAAATCTTGTGGA 3′	800 bp	[33]
		Dip1B	5′ ATGGCTTGTTTCGCTACATC 3′		
2	*cry4B*	Dip2A	5′ GGTGCTTCCTATTCTTTGG 3′	1293 bp	[33]
		Dip2B	5′ TGACCAGGTCCCTTGATTAC 3′		
3	*cyt1A*	Cyt1A1	5′ GTTGTAAGCTTATGGAAAAT 3′	701 bp	[34]
		Cyt1A2	5′ TTAGAAGCTTCCATTAATA 3′		
4	*cyt2*	Cyt2-1	5′ AATACATTTCAAGGAGCTA 3′	471 bp	[35]
		Cyt2-2	5′ TTTCATTTTAACTTCATATC 3′		
5	*cry11*	Cry11-1	5′ TTAGAAGATACGCCAGATCAAGC 3′	304 bp	[36]
		Cry11-2	5′ CATTTGTACTTGAAGTTGTAATCCC 3′		
6	*cry10*	Cry10-1	5′ ATATGAAATATTCAATGCTC 3′	614 bp	[37]
		Cry10-2	5′ ATAAATTCAAGTGCCAAGTA 3′		
7	*cyt1C*	Cyt1C1	5′ CAAAATCTACGGGAGCAAGG 3′	1320 bp	[23]
		Cyt1C2	5′ GGAAGGATCCCTTTGACTTTT 3′		
8	*p19*	P19-1	5′ GCAGGAGGAACATCACCATT 3′	291 bp	[23]
		P19-2	5′ GGATTTGCTGAGCAGGTCAT 3′		
9	*p20*	P20-1	5′ TGACGAGGAAACAGAGTATACGA 3′	704 bp	[23]
		P20-2	5′ TGAAAGGTTAAACGTTCCGATT 3′		
10	*parasporin1*	PS1-94F1	5′ AGCACCTAATGATGATAGAGGAA 3′	511 bp	[16]
		PS1-94R4	5′ CCCAGATTCAAATAATAACCAAGA 3′		
11	*parasporin2*	PS2-F	5′ GATGGTATTGCATTAAATAATGAAAC 3′	306 bp	[16]
		PS2-R	5′ TTCTCCACCAATTTCAAAGACT 3′		
12	*parasporin3*	PS3-F	5′ ATACAAGATGTGAGGAAATGATGA 3′	526 bp	[16]
		PS3-R	5′ GTATGGCTCAGCTCAATTTGA 3′		
13	*parasporin4*	PS4-F	5′ ACTAGTCAGCCTATAATCAGAACGA 3′	377 bp	[16]
		PS4-R	5′ ACTATTCCAGTACCAGTGTAACC 3′		
14	*parasporin5*	PS5-F	5′ TCAACGCCACAATTAACAAATA 3′	397 bp	[16]
		PS5-R	5′ TCCCTTGTATAGTTGCCTTTGT 3′		
15	*parasporin6*	PS6-F	5′ TGTTTACTATGTGAAAGGTGGAGA 3′	446 bp	[16]
		PS6-R	5′ CAATAGTGGTTCCTATTGGACC 3′		
